# Neural architecture of *Galathowenia oculata* Zach, 1923 (Oweniidae, Annelida)

**DOI:** 10.1186/s12983-016-0136-2

**Published:** 2016-02-08

**Authors:** Nadezhda N. Rimskaya-Korsakova, Alen Kristof, Vladimir V. Malakhov, Andreas Wanninger

**Affiliations:** Department of Invertebrate Zoology, Lomonosov Moscow State University, Leninskie Gory 1-12, 119234 Moscow, Russia; Department of Integrative Zoology, University of Vienna, Althanstraße 14, A-1090 Vienna, Austria

**Keywords:** Tubulin, Serotonin, FMRFamide, Medullary brain commissure, Medullary nerve cord, Single dorsal commissure, Unpaired ventral nerve cord, Pygidial somata clusters, Dorsolateral folds

## Abstract

**Background:**

Oweniids are marine tubeworms burrowing in muddy sediments that in current phylogenies form an early branching lineage within Annelida. Little is known about their general morphology, in particular the nervous system. Here we provide an immunocytochemical investigation of the nervous system of *Galathowenia oculata* in order to discuss putative ancestral neuronal features in Oweniidae.

**Results:**

Adult *Galathowenia oculata* have neither a supraesophageal ganglion nor ganglia associated with the ventral nerve cord. Instead, there is a dorsal brain commissure in the head collar that is engulfed by a cellular cortex. Accordingly, we herein term this neural structure “medullary brain commissure”. The anterior margin of the head collar exhibits numerous neurites that emerge from the brain commissure. The dorsolateral folds are innervated by the ventrolateral neurite bundles extending from the circumesophageal connectives. In the anterior uniramous and biramous segments immunoreactive somata are distributed evenly along the ventral nerve cord and arranged metamerically in the posterior-most short segments. One dorsal and two pairs of lateral neurite bundles extend longitudinally along the body. Numerous serially arranged circular neurite bundles were labeled in anteriormost long segments. Metameric arrangement of the circular neurite bundles stained against FMRFamide and acetylated α-tubulin is revealed in posterior short segments. For the first time immunoreactive somata arranged in clusters are reported within the pygidium in oweniids.

**Conclusions:**

Due to the lack of head appendages and a sedentary mode of life, *G. oculata* exhibits a single dorsal commissure (*versus* a brain with four commissures in most annelids). A “medullary brain commissure” is known so far only in Oweniidae and Echiura. Lack of ganglia and metamery in the ventral nerve cord of the anteriormost segments might be the result of the elongation of these segments. In the short posterior segments the metamery of immunoreactive somata and circular neurite bundles is conserved. We hypothesize that the unpaired ventral nerve cord in adult oweniids might be a result of an initially paired ventral nerve cord that fuses during development, a condition not uncommon within Annelida.

## Background

Oweniidae is a small annelid taxon with worldwide distribution from the continental slope to abyssal depths. They live in soft, muddy sediment and are very abundant in the Arctic Seas [1–3]. Currently, 55 valid oweniid species are known [3]. Oweniids deviate from other annelids in a number of morphological and developmental features, having non-typical spiralian embryogenesis [4], a unique mitraria larva with catastrophic metamorphosis [4, 5], deuterostome-like larval protonephridia [6], a monociliated epidermis in some species [7, 8], absence of a typical annelid-like cuticle [9, 10], absence of neuro-muscular synapses and control of locomotion through synapses on the surface of the extracellular matrix (ECM) [11, 12], as well as the absence of dissepiments separating the segments from each other [13].

The phylogenetic position of oweniids within Annelida is highly debated. Oweniidae was regarded as a taxon closely related to “archiannelids” [14–16], siboglinids [13, 17–20], or they were included in one clade together with Terebellida, Pogonophora and Sabellida [21]. Later, they were considered to be closest related to Sabellidae [22]. Molecular analyses of 18S and 28S rDNA sequences suggested a sister group relationship of *Owenia fusiformis* and *Apistobranchus typicus* (Apistobranchiidae) [23–25]. According to recent phylogenomic analyses, oweniids are considered one of the early branching annelid taxa, together with Magelonidae, Chaetopteridae, Sipuncula, and Amphinomidae [26–32].

So far, the anatomy of the oweniid nervous system is known mostly from one species, *Owenia fusiformis*, from histology and electron microscopy [11, 12, 33–37]. These studies revealed several peculiar features in the nervous system, including a single, non-ganglionated ventral nerve cord, a single brain commissure and a varying number of circular neurite bundles depending on segment size.

The present study investigates the morphology of the nervous system of the poorly studied oweniid species *Galathowenia oculata* (originally described as *Myriochele oculata*, [1, 13–15, 33, 34, 38, 39]) with immunocytochemical tools that provides data for morphological comparisons of the neural architecture of early branching annelids.

## Results

### Gross morphology of *Galathowenia oculata*

**Fig. 1:**
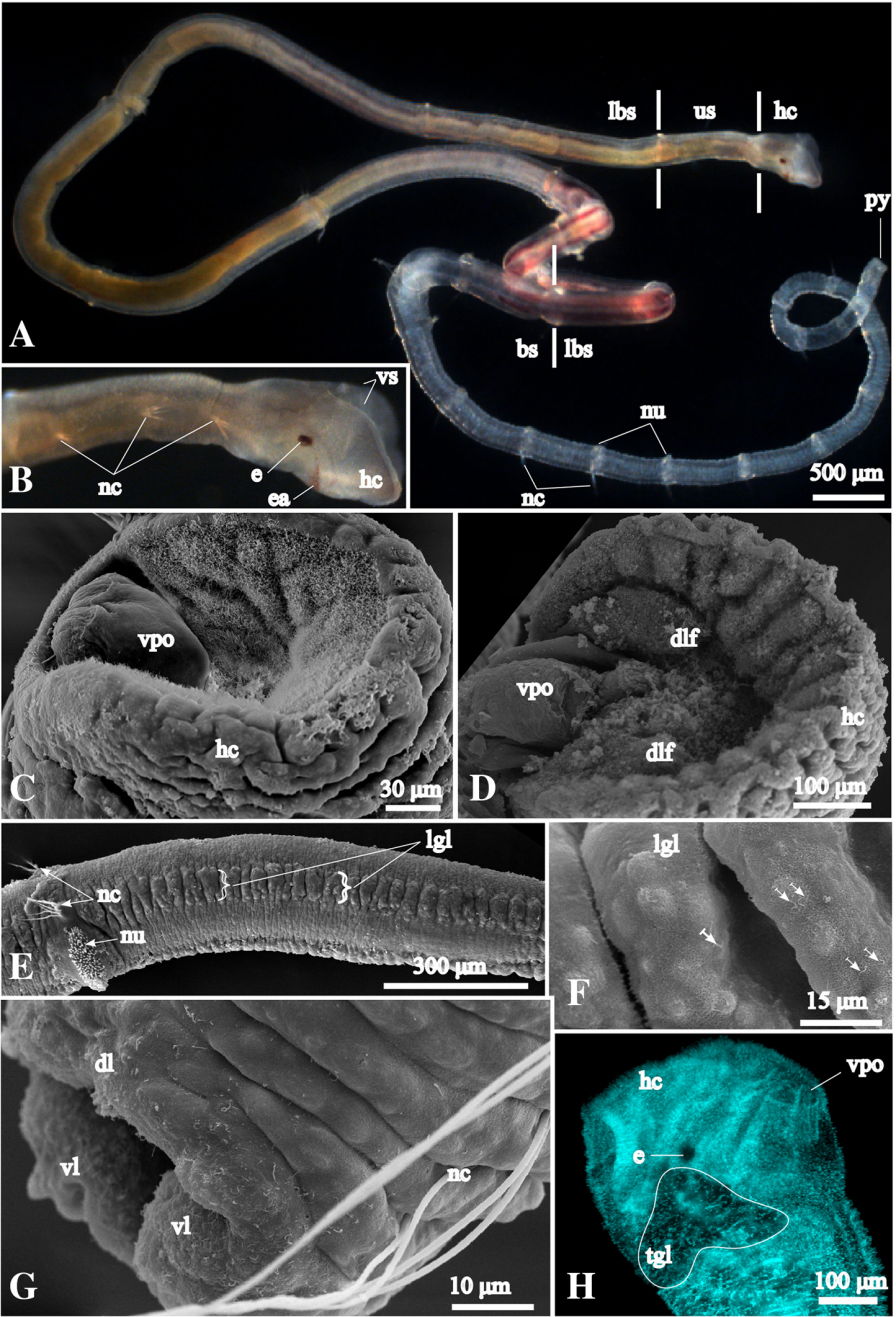
Gross morphology of *Galathowenia oculata*. **a**, **b** *G. oculata* without its tube (lateral view, ventral side is up). **a** Body divided into short uniramous, long biramous and short biramous segments bearing notopodial capillaries and neuropodial uncini. **b** Head collar with ventral slit, eyes and a pigmented arch between eyes. **c**, **d** Scanning electron micrographs (SEM) of the head collar, ventral side is to the left. The inner part is lined by cilia and bears the ventral pharyngeal organ. Note the variation of the absence (in **c**) and presence (in **d**) of dorsolateral ciliary folds. **e** SEM of one of the long biramous segments with the lateral glandular field, anterior end is to the left, ventral side is to the bottom. **f** Detail of glandular field with cilia (*arrows*) and gland papillae (*asterisks*), orientation as in (**e**). **g** SEM of the posterior-most 4 shortest segments and the pygidium with one dorsal and two ventral lobes, ventral side is to the bottom. **h** Nuclei labeling with DAPI (cyan) showing lateral view of head collar with ventral pharyngeal organ and an eye. Note that the absence of the cell nuclei indicates the position of the triangular glandular field. *bs* – short biramous segments, *dl* – dorsal pygidial lobe, *dlf* – dorso-lateral folds, *e* – eye, *ea* - pigmented arch between eyes, *hc* – head collar, *lgl* – lateral glandular field, *lbs* – long biramous segments, *nc* – notopodial capillaries, *nu* – neuropodial uncini, *py* – pygidium, *tgl* – triangular glandular field, *us* - short uniramous segments, *vl* – ventral pygidial lobes, *vpo* – ventral pharyngeal organ, *vs* – ventral slit

**Fig. 2:**
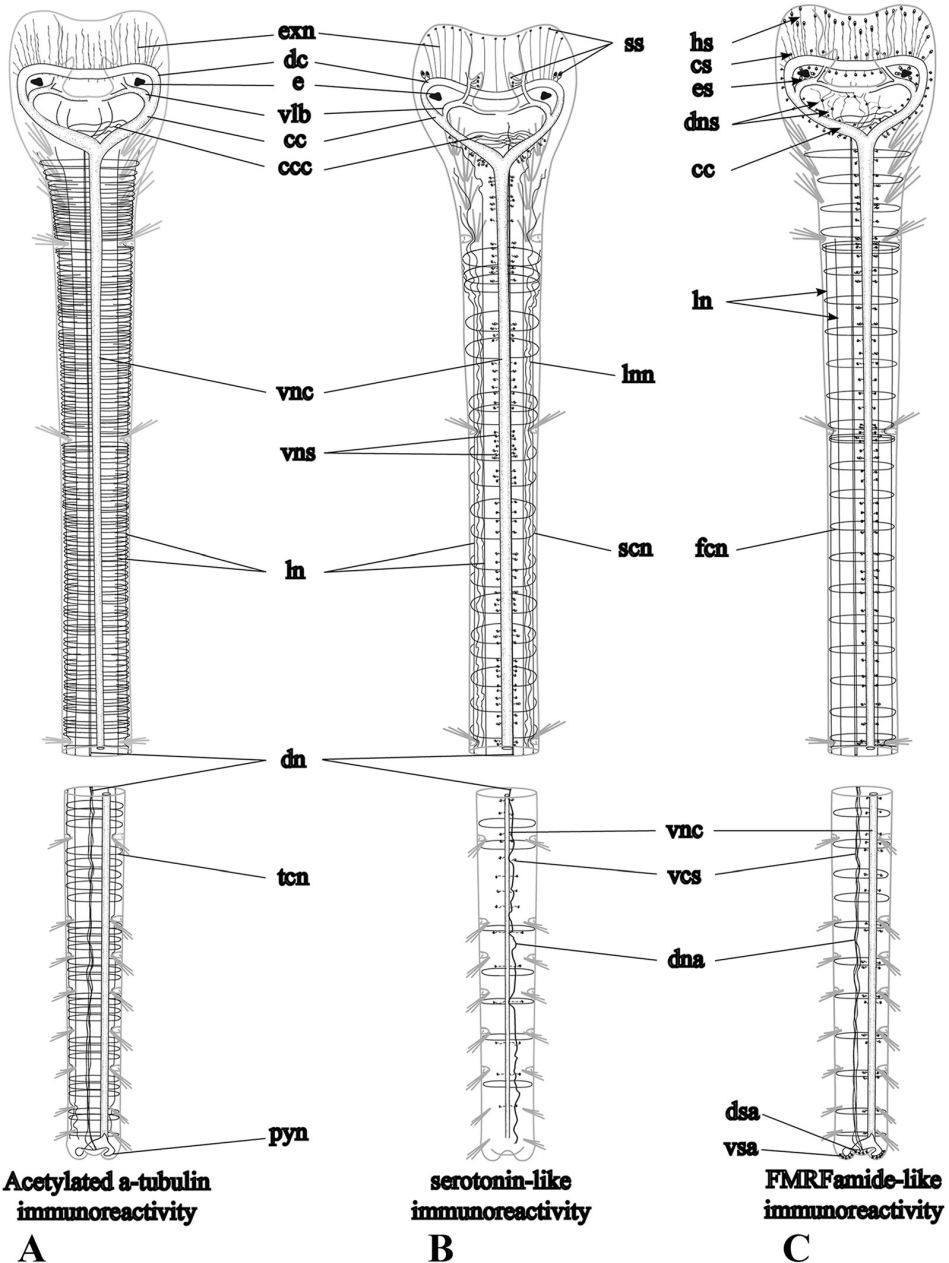
Schematic drawings of the general organization of the nervous system of *Galathowenia oculata*. (**a**) Tubulin-like, (**b**) serotonin-like and (**c**) FMRFamide-like immunoreactive nervous systems. Ventral views of anterior and posterior parts, nervous system shown in black, the body wall and chaetae are shown in grey. All three images show the same regions of the specimens, descriptions are given in the text. *cc* – circumesophageal connectives, *ccc* – transverse neurites interconnecting *cc*, *cn* – serially arranged circular neurite bundles, *dc* – dorsal commissure, *dn* – main dorsal neurite bundle, *dns* – immunocytochemically (ICC) positive somata associated with the main dorsal neurite bundle in head collar, *ds* – unipolar FMRFamide-lir somata associated with dorsal commissure, *dsa* – ICC-positive somata accumulation in dorsal pygidial lobe, *e* – eye, *es* – large ICC-positive somata around the eyes, *exn* – epidermal neurite plexus on outer side of head collar, *hs* – bipolar FMRFamide-lir somata located in head collar, *ln* – main lateral longitudinal neurite bundles, *lnn* – lateral neurite plexus between the lateral neurite bundles, *pyn* – pygidial neurites interconnecting the ventral and dorsal neurite bundles, *sc* – serotonin-lir cells of collar and dorsolateral folds, *ss* – serotonin-lir somata, *vcs* – serotonin-lir somata associated with ventral nerve cord, *vlb* – ventrolateral neurite bundle in head collar, *vnc* – ventral nerve cord, *vsa* – ICC-positive somata accumulations in ventral pygidial lobes

*Galathowenia oculata* inhabits slender tubes with two openings built by mud grains. The body of *G. oculata* is delicate; length and width are up to 30–40 mm and 0.5 mm, respectively (Fig. 1a). The prostomium and peristomium are fused, forming a head collar with a ventral slit (Fig. 1b). The head collar surrounds a midventral mouth and a ventral pharyngeal organ (Fig. 1c, d, h). Some of our specimens of *G. oculata* were observed to bear dorsolateral folds on the inner wall of the head collar (Fig. 1d). There are two ventro-lateral, red eyes and a pigmented arch between them in the outer wall of the head collar (Figs. 1b, h, 2a-c). The first three segments are equal in length, short and uniramous, exhibiting only notopodial capillaries (Fig. 1b), while the following 17 to 40 segments vary in length (Fig. 1a). The following nine anteriormost segments (4–12) are long and biramous and have notopodial capillaries and neuropodial uncini (Fig. 1a, e). Posterior segments are shorter, narrower and biramous (Fig. 1a). The posterior-most three to four segments are usually very short (30–50 μm), bearing one or two capillaries (Fig. 1g). The pygidium has one dorsal and two ventral lobes (Fig. 1g).

### Epidermal ciliation

**Fig. 3:**
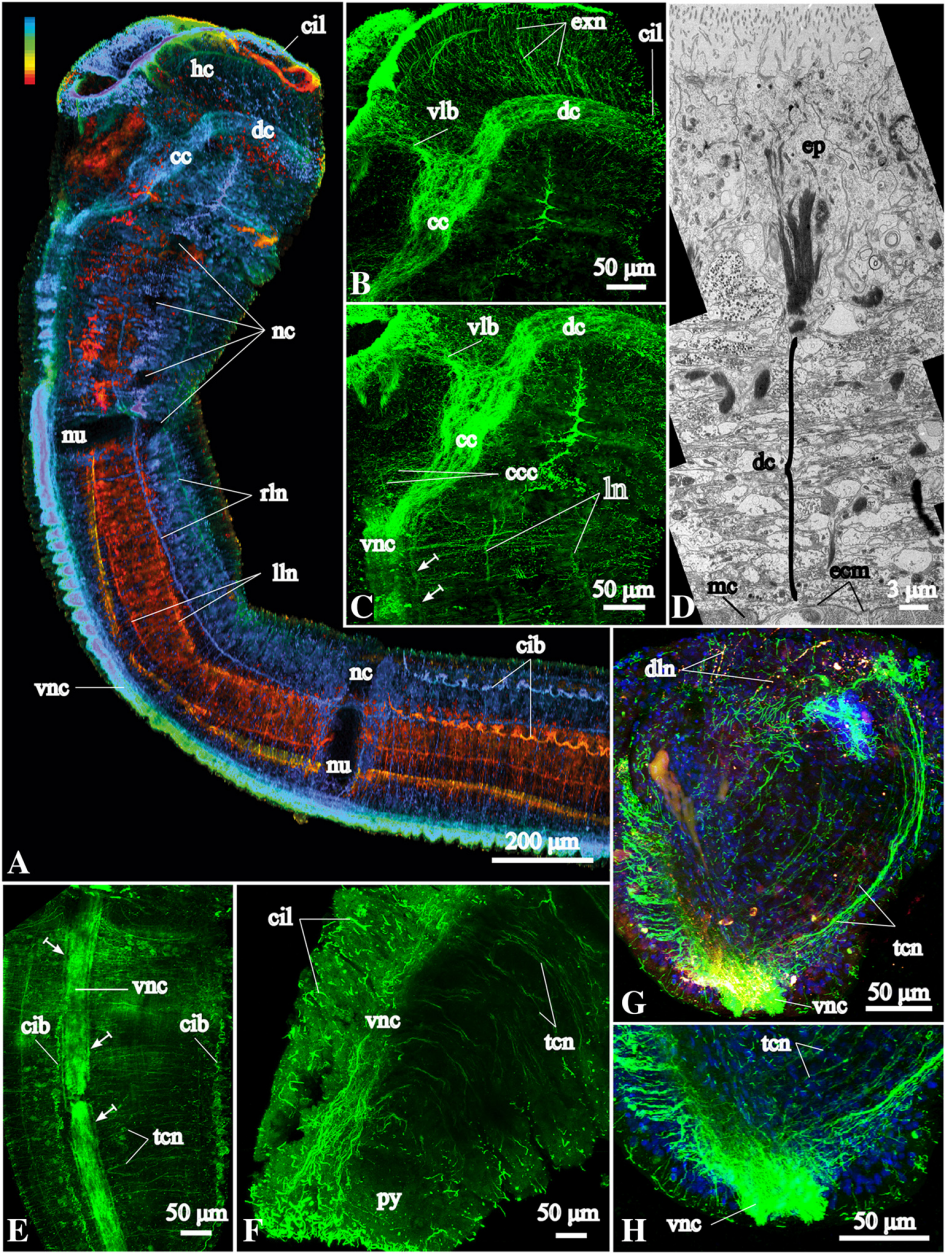
Tubulin-like immunoreactive (−lir) nervous system architecture in the head region and ultrastructure of the dorsal commissure of *Galathowenia oculata.* Labeling with antibodies against acetylated α-tubulin (green) and serotonin (red; shown only in **g**); nuclei labeling with DAPI (blue). Maximum projections and whole-amount specimens (**a**–**c**, **e**–**h**). TEM micrograph (**d**). **a** Color-coded image of the tubulin-lir dorsal commissure, ventral nerve cord and two pairs of the main peripheral longitudinal neurite bundles, lateral view, ventral side is to the left. **b**, **c** Lateral views show the nervous system within the head, note the collateral neurite bundles along the ventral nerve cord (arrows), ventral side is to the left. **d** Intraepidermal neurite bundles of the dorsal commissure (shown by bracket), transverse section of the head collar. **e** Tubulin-lir ventral nerve cord with collateral neurite bundles (arrows), trunk region; dorsal view, anterior is to the top. **f** Tubulin-lir ventral nerve cord in the posterior-most segments and pygidium, note cilia covering the surface, ventral side is to the top. **g**, **h** Tubulin-lir and serotonin-lir (in G) and only tubulin-lir (in H) ventral nerve cord with numerous serially arranged circular neurite bundles: transverse section of trunk region, note the paired structure of the nerve cord, ventral side is to the bottom. *cc* - circumesophageal connectives, *ccc* - neurites interconnecting *cc, cib* – ciliary band, *cil* – cilia, *dc* - dorsal commissure, *dln* – main dorsolateral neurite bundles, *dn* – main dorsal neurite bundle, *e* – eye, *ecm* – exctracellular matrix, *ep* – epidermis, *exn* – epidermal plexus within the outer side of *hc*, *hc* – head collar, *mc* – muscle cells, *py* - pygidium, *tcn* – tubulin-lir serially arranged circular neurite bundles, *vlb* – main ventrolateral neurite bundle, *vnc* – ventral nerve cord, *vpo* – ventral pharyngeal organ

The entire epidermis is covered by cilia as revealed by scanning electron microscopy (SEM) and acetylated α-tubulin-like immunoreactivity (−lir) (Figs. 1c–d, g, 3a, b, f). The surface of the inner walls of the head collar and the pygidial lobes is heavily ciliated (Fig. 1c, d, g). Additionally, there are several highly ciliated fields throughout the body. One of them overlies the dorsal commissure, a semicircular ciliary band on the dorsal side of the head (see Figs. 1b, 3b), and two lateral ciliary bands extending along the fifth chaetiger (Figs. 1f, 3a).

### Glandular fields

Glandular fields in the epidermis of *Galathowenia oculata* were revealed by autofluorescence. This was confirmed by ultrastructural data (not shown). Glands are concentrated into a pair of prominent triangular-shaped glandular fields behind the eyes (Fig. 1h). These fields expand backward to the dorso-lateral sides of the uniramous segments. Another two lateral glandular bands extend along the long biramous segments, from the fourth to the seventh, rarely exceeding the anterior part of segment eight (Fig. 1e, f). In the fifth segment, the lateral glandular bands extend along the lateral ciliary bands and together may serve as mucociliary tract (compare Fig. 1e, f). As the ciliary stripes reach the putative nephridal opening at the posterior part of the fifth segment (see Fig. 7h and [15], p.234) it is very likely that they serve excretory functions.

#### Nervous system architecture

The central nervous system (CNS) of *Galathowenia oculata* comprises a dorsal brain commissure, a pair of circumesophageal connectives and a ventral nerve cord (Figs. 2a–c, 3a). The peripheral nervous system (PNS) is represented by five main longitudinal neurite bundles and numerous serially arranged circular neurite bundles that together, with additional longitudinal neurite bundles, form an intraepidermal plexus. The majority of immunocytochemically positive (ICC-positive) somata is scattered along the ventral nerve cord. Parts of the PNS, along with the CNS, lie within the epidermis and include the circular and main longitudinal neurite bundles (Fig. 3d).

#### Neural elements of the head collar

**Fig. 4:**
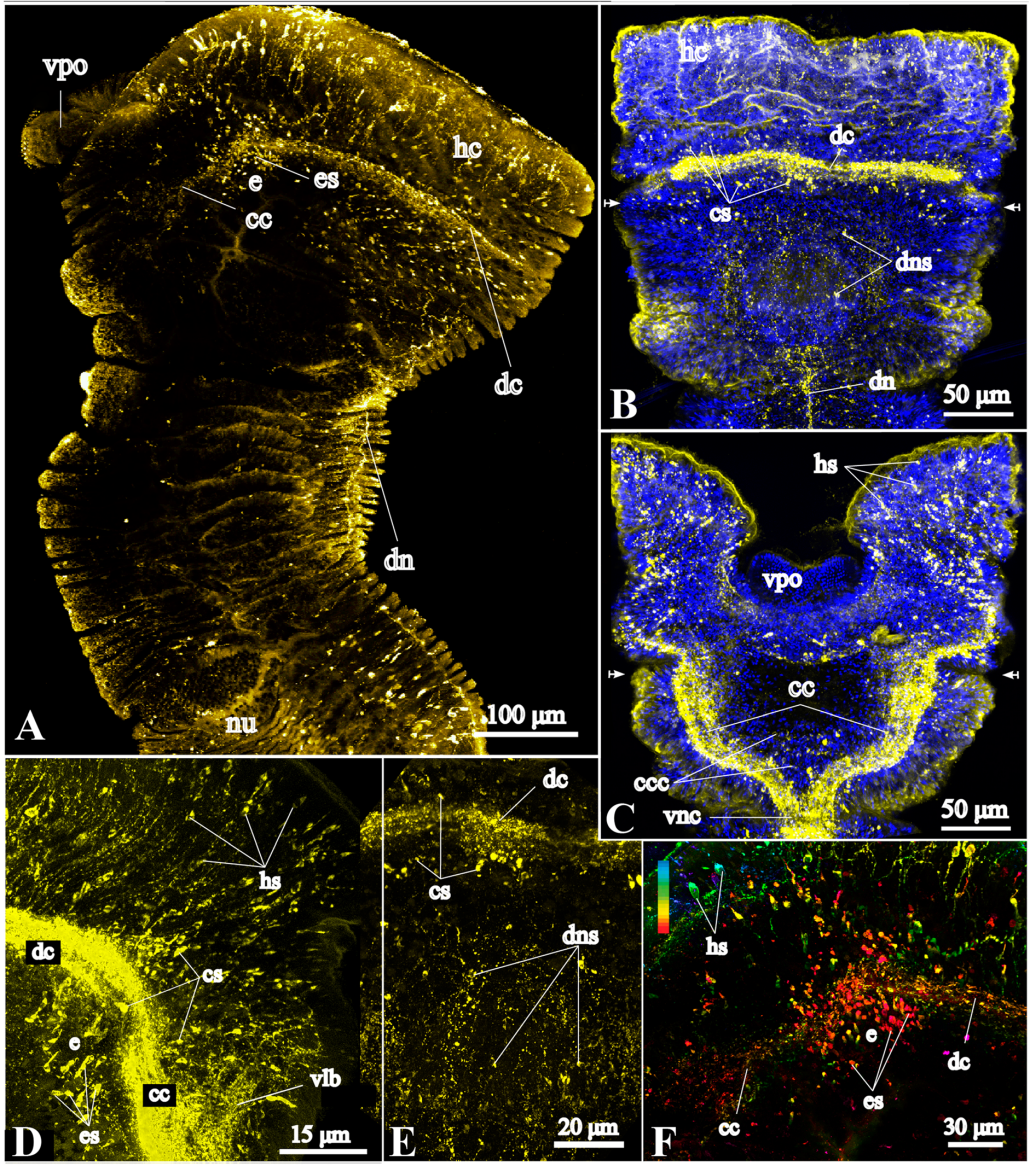
FMRFamide-like immunoreactive (−lir) nervous system in the head region. Anti-FMRFamide-lir (yellow) and nuclei labeling with DAPI (blue), maximum projections, whole-mount specimens. **a** Overview of FMRFamide-lir nervous system in the head region and the anterior-most segments, lateral view, ventral side is to the left. **b**, **c** FMRFamide-lir neural elements in the head region: the dorsal commissure and the circumesophageal connectives, dorsal and ventral views, respectively. Arrows indicate the position of eyes. **e** shows precise details of dorsal neural elements. **d** FMRFamide-lir somata of the head collar, lateral view. Immunocytochemically positive (ICC-positive) somata are distributed along the dorsal commissure within the head collar and around the eyes, ventral side is to the right. **e** Somata whose processes interconnecting the dorsal commissure and the main dorsal neurite bundle, dorsal view of the head collar (larger view of **b**). **f** Color coded image of ICC-positive somata around the eyes, lateral view of the head collar (the same specimen as in **a**). *cc* - circumesophageal connectives, *ccc* - neurites interconnecting *cc, cs* – ICC-positive somata along the dorsal commissure, *dc* - dorsal commissure, *dn* – main dorsal neurite bundle, *dns* – ICC-positive somata whose processes interconnect *dc* and *dn*, *e* – eye, *es* – large ICC-positive somata around the eyes, *hc* – head collar, *hs* – ICC-positive somata located in the epidermis of the anterior head region, *vlb* – main ventrolateral neurite bundle, *vnc* – ventral nerve cord, *vpo* – ventral pharyngeal organ

**Fig. 5:**
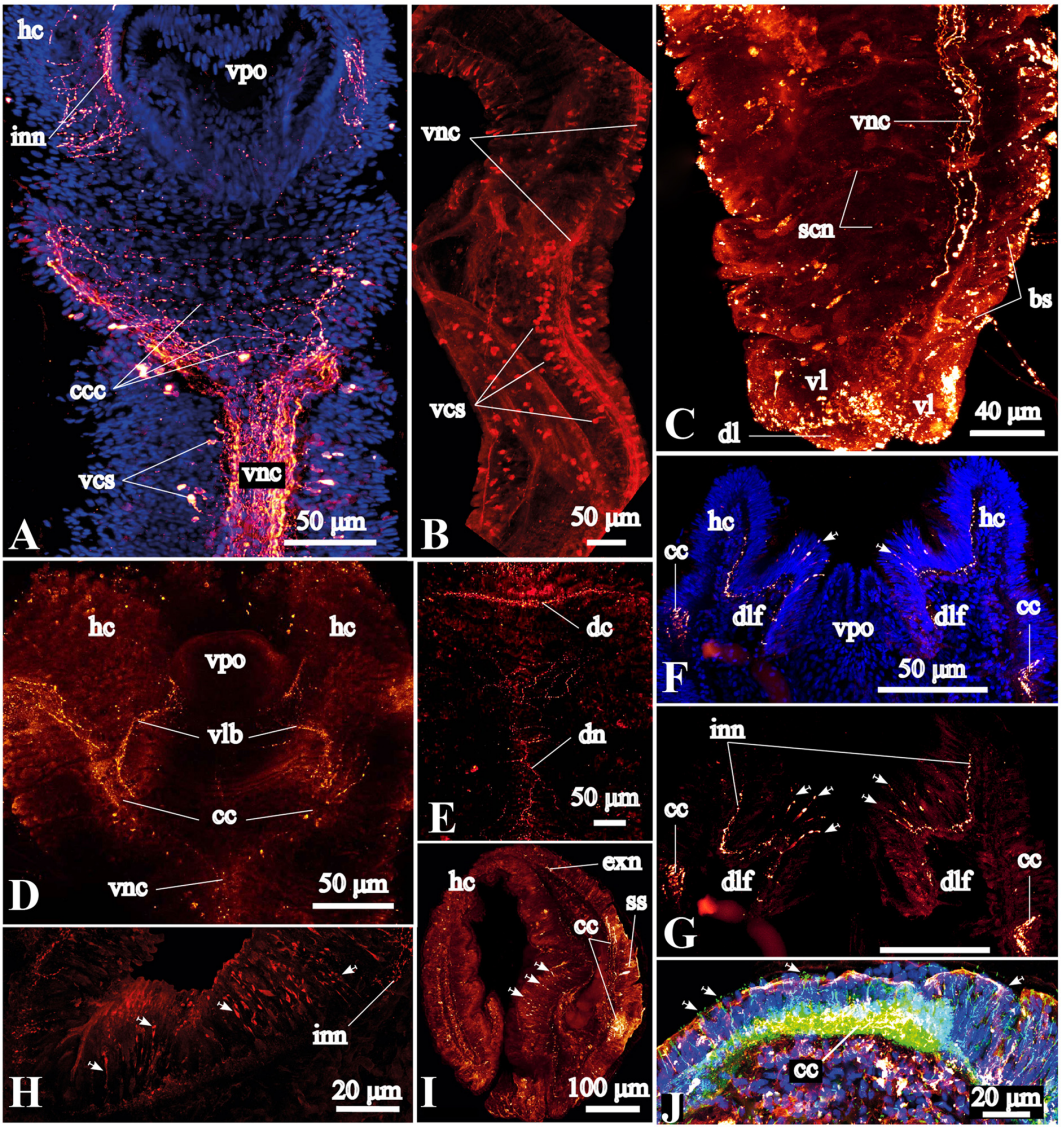
Serotonin-like immunoreactive (−lir) central nervous system of *Galathowenia oculata.* Labeling with anti-serotonin (*red*), anti-acetylated α-tubulin (green) and nuclear labeling with DAPI. Maximum projections (**a**, **b**, **f**–**j**) and optical sections (**c**–**e**) are shown. Whole-mount stained specimens (**b**–**e**), sectioned specimens with 100–200 μm thickness (**a**, **f**–**j**). **a** The serotonin-lir ventral nerve cord and circumesophageal connectives: frontal section, ventral view, anterior end is to the top. **b** The medullary organized ventral nerve cord, trunk region, ventrolateral view, anterior is to the top. **c** Serotonin-lir neurites of the ventral nerve cord in the posterior-most biramous segments, ventrolateral view, anterior is to the top. **d**, **e** Serotonin-lir elements of the head collar: ventral and dorsal views, respectively, anterior is to the top. **f**–**h** Serotonin-lir elements of the dorsolateral folds (*arrows*) and the inner wall of the head collar: frontal sections, ventral view, anterior is to the top. **i** Serotonin-lir elements (*arrows*) of the outer wall of the head collar: transverse section, ventral is to the bottom. **j** Sensory cells (*arrows*) in epidermis of the outer wall of the head collar: transverse section. *bs* – short biramous segments, *cc* – circumesophageal connectives, *ccc* – transverse neurites interconnecting circumesophageal connectives, *cn* – serially arranged circular neurite bundles, *dc* – dorsal commissure, *dl* – dorsal pygidial lobe, *dlf* – dorso-lateral folds, *dn* – main dorsal neurite bundle, *exn* – epidermal neurite plexus on outer side of head collar, *hc* – head collar, *inn* – neurite plexus within the inner side of the head collar epidermis, *lln* – main longitudinal neurite bundles of left body side, *nc* – notopodial capillaries, *nu* – neuropodial uncini, *rln* – main longitudinal neurite bundles of right body side, *scn* – serotonin-lir serially arranged circular neurite bundles, *ss* – serotonin-lir somata, *vcs* – serotonin-lir somata associated with the ventral nerve cord, *vl* – ventral pygidial lobes, *vnc* – ventral nerve cord

Labeling with antibodies against acetylated α-tubulin, FMRFamide and serotonin (5HT) equally reveals a loop formed by the dorsal brain commissure and the pair of circumesophageal connectives, the roots of which connect to the ventral nerve cord (Figs. 2a–c, 3a, 4a, 5a). The circumesophageal connectives are connected to numerous transverse commissures (Figs. 2a, b, 3c, 5a). In the head of *Galathowenia oculata,* numerous ICC-positive somata are present but do not form ganglia (Figs. 2b, c, 4b–d).

Anti-acetylated α-tubulin staining visualizes the massive dorsal commissure in the head collar approximately at the level of the eyes (Fig. 3a-c). It is 30 μm thick; the neurites within the dorsal commissure are organized evenly. Anti-acetylated α-tubulin staining revealed an extensive plexus of neurites that arise from the dorsal commissure anteriorly and run toward the external sides of the head collar (Fig. 3b). This tubulin-like immunoreactive (−lir) plexus comprises FMRFamide-lir and serotonin-lir neurites (see below and Figs. 4d, 5i). Laterally, the dorsal commissure projects into the circumesophageal connectives linked to each other via transverse neurites (Fig. 3c). The circumesophageal connectives loop and project to the ventral side where their roots unite with the ventral nerve cord (Fig. 3c). The middlemost part of the circumesophageal connectives is widest (75 μm in transverse section), from which a pair of broad ventrolateral neurite bundles splits off and projects toward the ventrolateral margin of the head collar (Fig. 3b, c). These bundles also include FMRFamide- and 5HT-lir neurites (see below and Figs. 4d, 5d). The ventrolateral neurite bundles give off neurite bundles that disperse in the epidermis of the internal sides of the head collar as well as the dorsolateral folds (Figs. 2b, 3b, 5d, f, g, h). The dorsal commissure and circumesophageal connectives give off numerous neurites that form a plexus in the epidermis of the outer side of the head collar (Figs. 3b, c, 4c, d). In the head collar epidermis there are numerous primary sensory cells with basal processes that form a plexus and connect to the dorsal commissure, connectives and ventrolateral bundles (Fig. 5j).

FMRFamide-lir neurites within the dorsal commissure and the circumesophageal connectives measure 20 μm in thickness and their distribution within the commissure is not even (Fig. 4a, b). The FMRFamide-lir somata are numerous in the head region and do not form accumulations; they lie along the dorsal commissure (*cs*), are scattered in the epidermis of the head region (*hs*), and around the eyes (*es*) (Figs. 2c, 4a–f). The ICC-positive somata that are localized in the outer wall of the head collar anteriorly to the dorsal commissure are bipolar cells with a diameter between 2 to 6 μm, while around the eyes the ICC-positive somata are large, elongated cells (6–7 × 2 μm) that are arranged in circular accumulations (Fig. 4d). The FMRFamide-lir dorsal commissure interconnects with the right and left circumesophageal connectives. The circumesophageal connectives give rise to a pair of neurite bundles that run to the ventral and ventrolateral sides of the head collar (Fig. 4d). The pair of FMRFamide-lir circumesophageal connectives interconnected by tiny transverse commissures projects into the ventral nerve cord (Fig. 4c).

Few anti-5-HT neurites are present in the dorsal commissure and the circumesophageal connectives (Figs. 2b, 5a, d, e, i). Fine commissural neurite bundles are present between the circumesophageal connectives; they are oriented transversely and ramify between each other (Fig. 5a). The serotonin-lir ventrolateral neurite bundles arise from each circumesophageal connective (Fig. 5d). Neurites from these ventrolateral bundles project toward the inner wall of the head collar (Fig. 5d). Numerous serotonin-lir cells are distributed in the epidermis of the collar and in the dorsolateral folds (Fig. 5f–i). These cells are connected to the neurite plexuses within the inner and outer part of the head collar body wall (Figs. 5a, g–i). The neurite plexus on the outer walls of the head collar has mostly connections to the circumesophageal connectives (Fig. 5i), while the neurite plexus on the inner wall of the head collar mostly connect to the ventrolateral neurite bundles (Fig. 5d, g, h). Relatively large serotonin-lir somata which are elongated (10–20 × 5 μm) are located along the circumesophageal connectives (Fig. 3i). Neither serotonin-lir nor FMRFamide-lir somata were found in the ventral pharyngeal organ.

### Ventral nerve cord

**Fig. 6:**
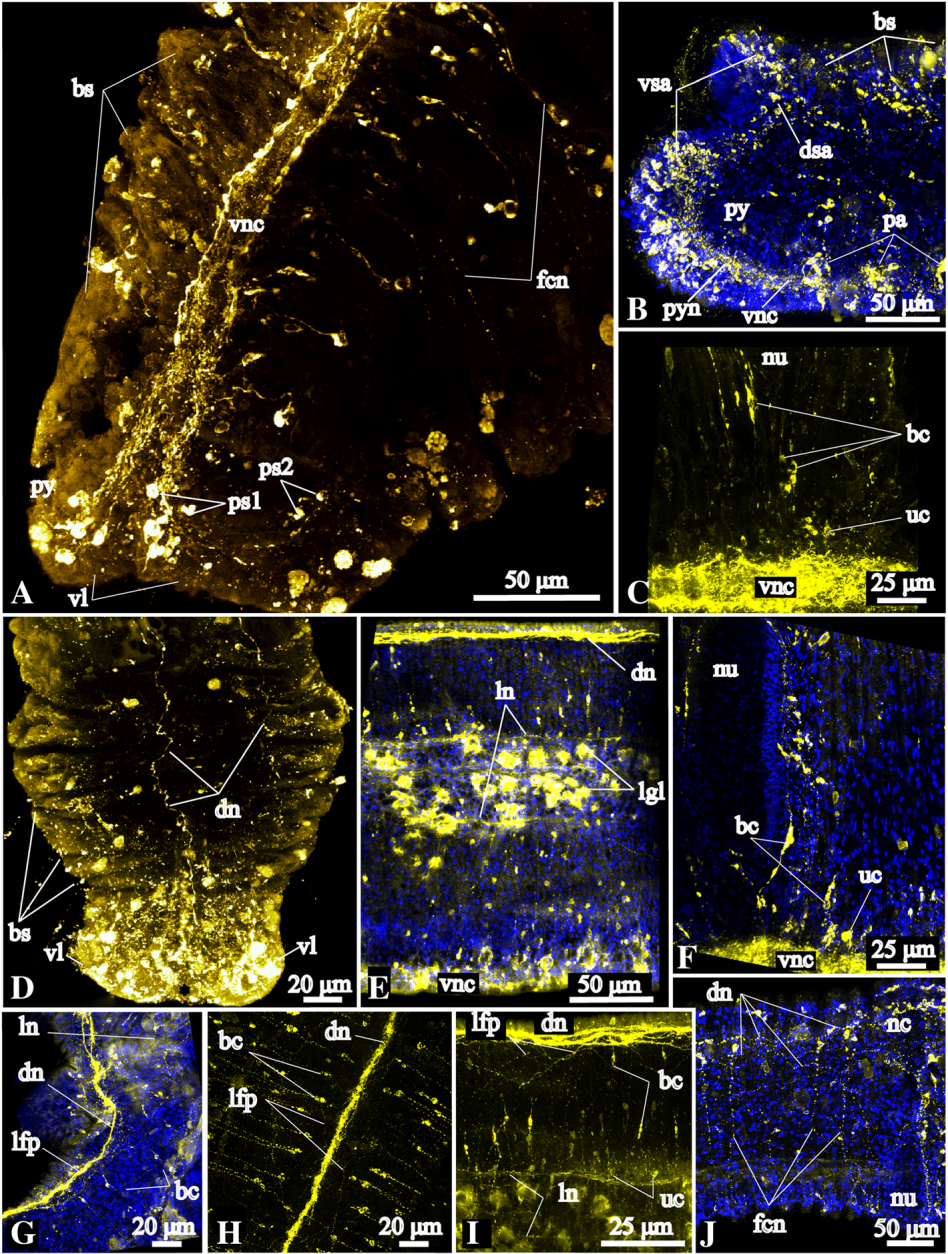
FMRFamide-like immunoreactive (−lir) nervous system in the trunk and pygidium of *Galathowenia oculata.* Anti-FMRFamide-lir (yellow), nuclei labeling with DAPI (blue), maximum projections, whole-mount specimens. **a** Ventral nervous system of the posterior-most segments and pygidium, ventrolateral view, anterior is to the top. Note the immunocytochemically (ICC) positive somata in the ventral and ventrolateral sides of the pygidium. **b** Pygidial ICC-positive somata accumulations, dorsolateral view of posterior-most chaetigers, anterior to the right. **c** Distribution of the ICC-positive somata associated with the ventral nerve cord, median portion of body, lateral view, anterior to the left. **d** Dorsal neurite bundles of the posterior-most segments, dorsal view, anterior to the top. **e** Ventral nerve cord and main peripheral longitudinal neurite bundles, left lateral view of anterior portion of body, anterior to the left. **f** ICC-positive somata associated with the ventral nerve cord, median portion of body, lateral view, anterior to the left. **g** Loose condition of the main dorsal neurite bundle in anterior segments, dorsolateral view, anterior to the bottom. **h** Main dorsal neurite bundle, serially arranged circular neurite bundles with associated bipolar cells and longitudinal neurites of the neurite plexus, dorsal view, anterior to the bottom. **i** Lateral view of main dorsal and lateral neurite bundles in the anterior body region, anterior to the left. Note the bipolar cells associated with the serially arranged circular neurite bundles. **j** Circular fibers of posterior-most segments, lateral view, anterior to the left. *bc* - bipolar cells, *bs* – short biramous segments, *dn* – main dorsal neurite bundle, *dsa* – ICC-positive somata accumulation in dorsal pygidial lobe, *fcn* – FMRF-amide-lir serially arranged circular neurite bundles, *nc* – notopodia capillaries, *nu* – neuropodial uncini, *lgl* – autofluorescing glands of lateral band, *lfp* – plexus of longitudinal neurites along main dorsal neurite bundle, *ln* – main lateral longitudinal neurite bundles, *pa* – ICC-positive somata accumulations in segments, *ps1-2* – ICC-positive somata of ventral and ventrolateral sides of pygidium, *py* – pygidium, *pyn* – pygidial neurite bundles interconnecting the ventral and dorsal neurite bundles, *uc* – unipolar cells, *vl* – ventral pygidial lobes, *vnc* – ventral nerve cord, *vsa* – ICC-positive somata accumulations in ventral pygidial lobes

Anti-acetylated α-tubulin, anti-5HT and anti-FMRFamide staining reveals the prominent single ventral nerve cord (Figs. 2a–c, 3a, f, 4c, 5a, b, 6a). All stainings showed that the ventral nerve cord extends from roots of the circumesophageal connectives, i.e., from the level of the first chaetiger until the posterior-most segment. A few neurites of the ventral nerve cord continue into the pygidium where they split into two bundles, projecting into the paired ventral pygidial lobes. These bundles run towards the dorsal side and connect to the main dorsal neurite bundle (see below). Neurites and ICC-positive somata of the ventral nerve cord labeled with various antibodies are organized in different ways.

Acetylated α-tubulin-lir shows that the transverse profile of the ventral nerve cord has two lobes, i.e., it constitutes a paired structure (figure-of-eight) (Fig. 3g, h). Besides, there are two collateral fine neurite bundles that run in parallel to the ventral nerve (Fig. 3c, e). FMRFamide-lir and serotonin-lir neurites of the ventral nerve cord are not paired and are not present in the collateral bundles (compare Figs. 3c with 4c and 5a).

The FMRFamide-lir elements of the ventral nerve cord are distributed along the entire length of the worm including the posterior-most segment (Figs. 2c, 6a–c, e, f). Two neurite bundles of the ventral nerve cord extend into the pygidium (Fig. 6a, b). Somata labeled with antibodies against FMRFamide are located pairwise along the left and right sides of the ventral nerve cord (Fig. 6a, c, f). Most of the ICC-positive somata are small (4–5 μm) and have a drop-like shape; other somata are large, around 9–11 μm in diameter. In the posterior (short) segments ICC-positive somata form accumulations in the anterior parts of each segment (Fig. 6b).

**Fig. 7:**
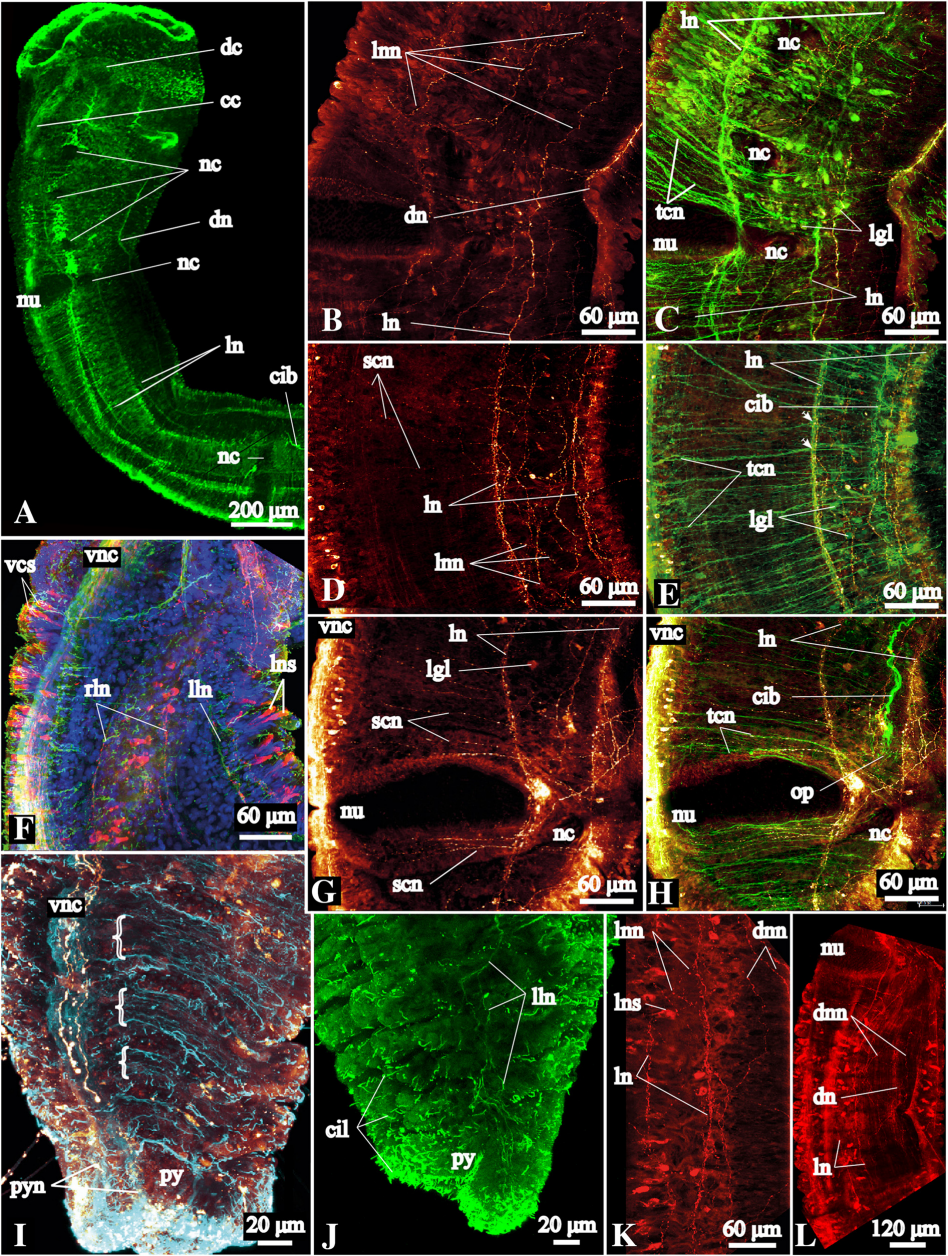
Peripheral nervous system in the trunk of *Galathowenia oculata.* Anti-acetylated α-tubulin (green, cyan) and anti-serotonin (red) immunoreactivity (lir) nuclei labeling with DAPI (blue), maximum projections, whole-mount specimens. Ventral to the left, anterior to the top. **a** Main tubulin-lir longitudinal neurite bundles up to the 5^th^ chaetiger, dorsolateral view. **b** Serotonin-lir lateral neurite plexus in the 2^nd^, 3^rd^ and 4^th^ chaetigers, lateral view. **c** The same fragment as in B. Main tubulin-lir lateral longitudinal neurite bundles and numerous serially arranged circular neurite bundles. **d**, **e** Serotonin-lir and tubulin-lir circular neurite bundles and plexuses between the main longitudinal neurite bundles, the 5^th^ chaetiger, lateral view. Arrows point to ramifying of neurite bundles. **f** Serotonin-lir somata of the ventral nerve cord and main longitudinal neurite bundles, the 6^th^ chaetiger, lateral view. **g**, **h** Serotonin-lir and tubulin-lir circular neurite bundles in the region of the parapodia, posterior portion of the 5^th^ chaetiger, lateral view. **i** Serotonin-lir and tubulin-lir circular neurite bundles in the posterior-most segments (brackets mark tubulin-lir neurite bundles in each segment), ventrolateral view. **j** Tubulin-lir longitudinal neurite bundles in the posterior-most segments, lateral view. **k** Serotonin-lir somata associated with the neurite plexus and the main lateral longitudinal neurite bundles, lateral view. **l** Main serotonin-lir dorsal neurite bundle associated with the dorsal neurite plexus, dorsolateral view. *cc* – circumesophageal connectives, *cib* – ciliary bands, *cil –* cilia, *dc* – dorsal commissure, *dn* – main dorsal neurite bundle, *dnn* – dorsal neurite plexus, *lgl* – autofluorescent glands of lateral band, *lln* – left main lateral neurite bundle, *ln* – main lateral neurite bundle, *lnn* – lateral neurite plexus, *lns* – immunopositive somata associated with the main lateral neurite bundles, *nc* – notopodia capillaries, *nu* – neuropodial uncini, *op* – nephridiopore, *py* – pygidium, *pyn* – pygidial neurite bundles interconnecting the ventral and dorsal neurite bundles, *rln* – right main lateral neurite bundle, *scn* – serotonin-lir serially arranged circular neurite bundles, *tcn* – tubulin-lir serially arranged circular neurite bundles, *vcs* – serotonin-lir somata associated with ventral nerve cord, *vnc* – ventral nerve cord. Legend for cover image: Medullary brain commissure of *Galathowenia oculata* (Oweniidae; Annelida) showing cell nuclei by DAPI staining (blue) and FMRFamide-like immunoreactivity (yellow)

Serotonin-lir neurites in the ventral nerve cord are numerous in the anterior segments and their number decreases in the posterior short segments (Figs. 2b, 5b, c). Few neurite bundles reach the posterior-most segment. Serotonin-lir somata are scattered continuously bilaterally arranged by pairs along the ventral nerve cord (Figs. 5b, 7f). There are around 6–8 pairs of ICC-positive somata in the short uniramous segments. In the biramous segments, there are 50–100 pairs of somata within the long segments and 6–8 pairs per short segment. ICC-positive somata are large and elongated (16–20 μm in length). There are no ganglion-like structures along the ventral nerve cord, but there are gaps between accumulations of the serotonin-lir somata (for example, at the levels of the neuropodia; Fig. 7g).

### Main peripheral longitudinal neurite bundles

In *Galathowenia oculata* there are five main peripheral longitudinal neurite bundles: an unpaired dorsal one and two pairs of lateral ones running along the lateral glandular bands and notopodial chaetae (Figs. 2a–c).

Five tubulin-lir main peripheral longitudinal neurite bundles are present in the first uniramous segment and give off short branches into the peristomium (Figs. 2a, 7a, c, e, h, j). The lengths of the main longitudinal neurite bundles differ (Fig. 2a): the main dorsal longitudinal neurite bundle (4–5 μm in thickness) extends into the pygidium. Two pairs of the main lateral tubulin-lir neurite bundles run along both sides of the notopodial chaetae (Fig. 7c); in the long biramous segments they run along both sides of the glandular bands and the ciliary bands (Figs. 7a, e, h). The ventro-lateral neurite bundles are more prominent (8 μm in diameter) than the dorso-lateral pair (4 μm in diameter). Few lateral tubulin-lir neurite bundles are visible in the posterior-most segments (Fig. 2a, 7j).

FMRFamide-lir neurite bundles were revealed within each of the main longitudinal tubulin-lir neurite bundles, the unpaired dorsal and the two pairs of lateral bundles (Figs. 2c, 4a, b, 6e). The main dorsal FMRFamide-lir neurite bundle runs from the first chaetiger into the pygidium (Figs. 4a, b, 6b, d). The diameter of the FMRFamide-lir dorsal neurite bundle varies between 1 and 5 μm. In the uniramous and anterior biramous segments the FMRFamide-lir dorsal neurite bundle is wider, loose, and often branched (Fig. 6g, i). There are numerous additional longitudinal neurites on both sides of the dorsal neurite bundle. These neurite bundles ramify, join the main lateral neurite bundles and have connections with the circular neurite bundles, thereby forming an intraepidermal neurite plexus (Fig. 6g–i). The middlemost part of the main dorsal neurite bundle is thinner and denser (Fig. 6h) than in the anterior part of the worm; posteriorly it is represented by individual widely spaced neurite bundles (Fig. 6j). ICC-positive somata are present along the entire length of the main dorsal neurite bundle and in the dorsal peristomium (Figs. 4b, e, 6g–i). The main dorsal neurite bundle in particular includes the neurites of bipolar FMRFamide-lir cells (Fig. 6h). The main dorsal neurite bundles as well as numerous tiny neurites from the dorsal part of the intraepidermal plexus in the peristomium have connections to the dorsal brain commissure (Figs. 2c, 4b, e). FMRFamide-lir main lateral longitudinal neurite bundles were found along the glandular band (Figs. 1e, 6e, i). The main lateral FMRFamide-lir neurite bundles are equal in diameter (compare with the serotonin-lir ones), around 1–2 μm, and do not split. FMRFamide-lir unipolar and bipolar cells are associated with the main lateral neurite bundles (Fig. 5i).

Serotonin-lir was also revealed in the five main peripheral longitudinal neurite bundles (Figs. 2b, 7d, f, k). The unpaired main dorsal neurite bundle (3 μm) gives off some neurites into the head (mostly in the peristomium) which ramify there into the plexus (Fig. 5e); posteriorly, these neurites form the main dorsal neurite bundle that extends along the length of the body (Figs. 2b, 7b, l). Somata labeled with antibodies against 5HT were not found in the main dorsal neurite bundle. There are two pairs of main lateral longitudinal neurite bundles extending from the fourth chaetiger (Fig. 7b) and terminating at different levels. The dorsolateral pair extends until the end of the eighth chaetiger, while the ventrolateral pair extends to the middle part of the ninth chaetiger. At the anterior and posterior ends of the lateral neurite bundles there are plexuses of numerous neurites projecting in different directions (Figs. 7b). Serotonin-lir unipolar cells spread along the main lateral longitudinal neurite bundles are large, 12 μm in length, and conical (they have wide apical parts and narrow basal parts that give off neurites) (Fig. 7f, k).

### Circular neurite bundles and plexuses

Anti-α-tubulin immunofluorescence shows regularly arranged circular neurite bundles extending from the ventral nerve cord to the main longitudinal neurite bundles (Figs. 2a, 7c, e, h). The number of the bundles increases with the length of the segment. Accordingly, there are about six to seven neurite bundles per short uniramous segment, while there are up to 60 neurite bundles per long biramous segment. Thick serially arranged bundles are semicircular; they extend from the ventral nerve cord to the main lateral longitudinal neurite bundles. As they reach the main ventrolateral longitudinal neurite bundles they split into two to three finer neurite bundles that extend to the main dorsolateral longitudinal neurite bundles (Fig. 7e). These circular neurite bundles underlie the bands of cilia and glands (Figs. 1e, 7c, e). A few fine circular neurite bundles extend to the main dorsal neurite bundle. In posterior short segments, circular tubulin-lir neurite bundles exhibit the tendency to concentrate (Figs. 2a, 7i). Surrounding the noto- and neuropodia there are tiny circular tubulin-lir neurite bundles (Fig. 7h).

FMRFamide-lir circular neurite bundles are abundant and associated with regularly spread bipolar cells (6 μm; Figs. 2c, 6a, c,f–j). Most FMRFamide-lir bipolar cells lie posterior to the parapodia (Figs. 6c, f). From the head to the pygidium the number of the neurite bundles per chaetiger decreases (Figs. 2c, 6a, h).

Serotonin-lir circular neurite bundles are arranged irregularly (Fig. 7d, g). They do not form bundles, except around the parapodia (Fig. 7g). Serotonin-lir somata are found associated with the ventral nerve cord and with the main lateral longitudinal neurite bundles, but not with the dorsal one (Fig. 7f, k, l). Serotonin-lir neurites of the lateral (Fig. 7k) and dorsal (Fig. 7l) longitudinal neurite plexuses show longitudinal, transverse and oblique orientations.

### Pygidial neural elements

The ventral nerve cord revealed by tubulin-lir extends to the pygidium and splits into two bundles, which then turn dorsally and join the main dorsal neurite bundle. FMRFamide-lir neurites of the ventral nerve cord project into the pygidium and divide into two branches that run into the ventral pygidial lobes, then around the anus and to the dorsal side, where they connect to the main dorsal longitudinal neurite bundle (Figs. 1c, 6a, b). The pygidium exhibits a vast number of FMRFamide-lir somata. Some of them are associated with the ventral nerve cord and lie either close to or at the lateral sides of the pygidium (Fig. 6a). Moreover, within the pygidium there are three accumulations of large somata (6–8 μm) lying close to each other in the epidermis and adjacent to the pygidial neurite bundles (vsa, Fig. 6b). Around 60 somata are accumulated in each ventral pygidial lobe. The third accumulation contains 10–20 somata in the dorsal lobe (dsa, Fig. 6b) of the pygidium, which is heavily ciliated (Fig. 1g). We did not find any serotonin-lir elements in the pygidial lobes.

## Discussion

### Central nervous system gross architecture of *Galathowenia oculata*

Our study is the first investigation of the nervous system in a representative of Oweniidae using immunocytochemical methods in combination with confocal laser-scanning microscopy, a method proven to be effective for the study of the nervous systems in numerous invertebrate taxa including annelids [40–42]. An intraepidermal position of the nervous system, as in *G. oculata*, has been regarded as the plesiomorphic state for Bilateria [41, 43–45]. Such a basiepithelial position is found in many annelid groups [41, 43, 46]. An intraepidermal CNS is also characteristic for the oweniids and their putative sister group, the magelonids [3, 12, 35, 47, 48] and we therefore suggest that it was also present in the last common ancestor of Oweniidae.

### Medullary brain commissure

In general, the supraesophageal ganglion of annelids comprises a compact central mass of neuropil surrounded by a cell cortex [49]. Our study did not reveal a ganglionic organization around the dosal commissure in *Galathowenia oculata*. Serotonin-lir and FMRFamide-lir somata do not form a compact cell cortex and tubulin-lir neurite bundles do not form a swelling within the dorsal commissure (Figs. 2a-c, 4a, b, 5d, i). Few pairs of serotonin-lir somata were found on the lateral sides of the dorsal commissure. FMRFamide-lir unipolar somata are numerous and uniformly distributed along the loop of the dorsal commissure and the circumesophageal connectives. Nerve cords or neurite bundles that are surrounded by a cellular cortex are commonly termed medullary nerve cords/bundles [49]. This conforms to the situation found in the oweniid dorsal brain commissure analysed herein and we therefore use the term “medullary brain commissure” for the respective structure. A similar organization of the neurite loop without a ganglion-like thickening is known from Echiura, where along the neurite loop of *Bonellia viridis* and *Urechis caupo* numerous FMRFamide-lir somata and several pairs of serotonin-lir somata were found [40, 50–52]. So far, there is not enough data for explanation of the absence of a distinct supraesophageal ganglion and presence of the only thick commissure in both taxa, Oweniidae and Echiura. For example, it might be due to a similar way of deposit feeding, or overall sedentary lifestyle, or developmental processes etc. Also, this data has to be confirmed by transmission electron microscopy or histology-based studies, since labeling of FMRFamides and serotonin only visualizes those parts of the nervous system that contain the respective neuroactive substances [46].

### Number of brain commissures

The presence of four transverse commissures has been considered as the most conservative feature of the adult brain in Annelida [41, 53]. Staining against acetylated α-tubulin has shown that there is only one thick brain commissure in *Galathowenia oculata* (Figs. 2a, 3b). Bubko and Minichev [34] showed that *Owenia fusiformis* has two commissures; a main brain commissure and an additional minute supraesophageal commissure that supposedly emerges from the nuchal organ. No nuchal organ was found in any of our specimens of *G. oculata*. Capa et al. [3] also stated the absence of nuchal organs in this taxon in their taxonomic revision of Oweniidae. Accordingly, the additional commissure found in *Owenia fusiformis* may emanate from the tentacles. Species from the genus *Owenia* have a pronounced tentacle crown serving for gas exchange and collection of food particles as well as particles for tube building. In species of the genus *Galathowenia* there are no tentacles [3], and collection of particles is performed with the ventral pharyngeal organ (which is absent in *Owenia*) and the dorsolateral folds of the collar (see below and Fig. 1c, d).

A single supraesophageal commissure can be found in various tubicolous and burrowing annelids including Pectinariidae, Terebellidae, Ampharetidae, Chaetopteridae [41], Arenicolidae [54], Maldanidae [55] and Echiura [40, 50–52]. All of them share a combination of behavioral features as burrowing sediment dwellers, resting inside their tubes/burrows and feeding on sediment deposits. Probably due to these habits there is no demand to form numerous diverse head appendages with sensory organs requiring well-developed transverse commissures and this might be the reason why there is only a single dorsal commissure in *G. oculata*.

Nonetheless, in all annelids that have ever been regarded as a sister clade to Oweniidae (e.g., “archiannelids”, Apistobranchiidae, Sabellida, Siboglinidae, Terebellida, Magelonidae) there is no similar organization of a brain commissure as a single neurite loop. The group of errant deposit-feeders previously known as “archiannelids” exhibits a well-developed ganglion and a prominent brain neuropil (e.g., Protodrilidae, Saccocirridae, Dinophilidae and Nerillidae) or have two roots of circumesophageal connectives (e.g., Polygordiidae) [56–61]. Sedentary filter-feeding annelids such as Sabellidae, Sabellariidae and Serpulidae have four supraesophageal commissures [41, 62]. Evidently, the complex brain structure of sedentary annelids can be explained by the presence of the various appendages involved in filtration. In Siboglinidae there is a complex brain with several commissures [63–67]. In vestimentiferan siboglinids there is a prominent brain including a subesophageal and a supraesophageal ganglion with two transverse commissures. A brain with such complexity corresponds to their large tentacle apparatus [64, 66, 68]. In tiny frenulate siboglinids there is an additional tentacular neurite commissure [63, 65], and even in dwarf males of *Osedax* there are two commissures [67]. Magelonidae, Orbiniidae, Flabelligeridae, Apistobranchidae, Poecilochaetidae, Trochochaetidae, Scalibregmatidae and Paraonidae all exhibit four brain commissures that likely correspond to a mode of life as errant sediment burrowing annelids that are deposit or suspension feeders [41, 62, 69, 70]. Thus, the number of brain commissures in adult annelids is largely a reflection of their mode of life that can hardly be used for phylogenetic inferences.

### Dorsolateral folds and head collar innervation

For the first time the innervation of the dorsolateral folds and the anterior margin of the head collar of *Galathowenia oculata* are described (Figs. 2b, g, h, j, l–o, 6a–c). The head collar contains numerous serotonin-lir and FMRFamide-lir somata, while the dorsolateral folds contain somata labeled with antibodies against serotonin. Both the head collar and dorsolateral folds are covered by cilia and are involved in sediment particle sorting. The newly revealed somata in these organs might suggest that they are involved in sensory perception, which is known from ciliated sensory cells in tentacles in a number of annelids [56, 71–73]. The ventrolateral neurite bundles include neurites in the head collar and the dorsolateral folds (Figs. 2, 3b, 4d, 5a, d, f-h). To date, similar neurite bundles branching off from circumesophageal connectives have not been shown in other annelids.

### Medullary ventral nerve cord

According to our immunocytochemical stainings, the ventral nerve cord of *Galathowenia oculata* is medullary organized; FMRFamide-lir and serotonin-lir somata are scattered evenly along the anterior part of the ventral nerve cord (Figs. 2b, c, 5b, 6e). There are neither distinct ganglia nor swellings in the nerve cord of *G. oculata*. Although the latter were found in *Owenia fusiformis* in the fourth and ninth segments, this was explained by the presence of complex dissepiments serving as sphincters [14, 34].

A medullary ventral nerve cord is common in several annelids belonging to different phylogenetic lineages: *Polygordius*, *Chaetogordius*, *Saccocirrus*, *Protodrilus,* Oweniidae, *Magelona*, Siboglinidae, Echiura, some species of Opheliidae and Maldanidae [40, 43, 47, 51, 68, 74]. A ventral nerve cord with ill-defined ganglia is also known for some oligochaetes such as Naididae or Enchytraeidae or some Lumbricidae [43, 45, 75–79]. Most of the taxa mentioned above are regarded as either specialized interstitial meiofaunal animals [80–82] or sedentary sediment feeders [3, 40, 55, 74].

In long segments of *G. oculata* FMRFamide-lir somata are distributed evenly, whereas in short posterior segments somata tend to accumulate (compare Fig. 6b and 6e). A similar situation is found in Siboglinidae. For instance, in frenulate siboglinids ganglia of the ventral nerve cord are present in short segments of the opisthosoma, whereas in the very long segments of the trunk and the forepart the ventral nerve cord is medullary [74]. Likewise, Protodrilidae, Polygordiidae and Maldanidae have elongated segments and a medullary condition of the ventral nerve cord [43, 46, 55]. Accordingly, the medullary organization of the ventral nerve cord described herein for *G. oculata* might be due to the elongation of short segments that might have occurred in the last common oweniid ancestor. Very likely, the elongation of segments emerged in different lineages of annelids due to their particular life history or ecology (e.g., sessile or interstitial). Whether or not a medullary nerve cord is only found in representatives that have long segments remains to be shown by future investigations.

### Unpaired ventral nerve cord

During development of the oweniid mitraria larva two separate ventral neurite bundles fuse to a single one [5]. Adult *Galathowenia oculata* show a profile of the ventral nerve cord that appears figure-eight-shaped along its entire length (Figs. 3g, h). We hypothesize the fusion of both cords in *G. oculata*. Transformation of a widely separated pair of ventral neurite bundles into a single cord during larval development is shown for instance in other Oweniidae, Sipuncula, and Echiura [5, 40, 42, 52]. Therefore, it appears plausible that the dineuronal organization of the ventral nerve cord is an ancestral feature for Oweniidae as it has been already suggested for all annelids [43], although there are other views [53].

There are two widely separated neurite bundles, the circumesophageal connectives, in *G. oculata* (Fig. 2) and other Oweniidae [3]. These are also visible in the second anterior segment of siboglinids [64, 66, 68, 83, 84], which were considered as sister group to Oweniidae [18]. Also, there are paired ventral neurite bundles in Mageloniidae and Chaetopteridae [47, 48]. In vestimentiferan siboglinids, two neurite bundles interconnect the subesophageal ganglia and the ventral nerve cord [68, 84]. On the other hand, in Magelonidae, Oweniidae and Chaetopteridae, these neurite bundles are the circumesophageal connectives and most likely homologous to each other [34, 47, 48]: they unite at the level of the first setiger in oweniids (Fig. 2) [3], the ninth setiger in *Magelona sp.* [47] and the twelfth setiger in *Chaetopterus variopedatus* [48]. This is one of the morphological features shared between oweniids, magelonids and chaetopterids, but not with the siboglinids.

### Peripheral nervous system

Five main peripheral longitudinal neurite bundles are known from *Owenia fusiformis* [34] and are also reported herein for *Galathowenia oculata* (Fig. 2). The most prominent main neurite bundle is the dorsal one. For the first time the dorsal neurite bundle is found to have a connection to the CNS in oweniids. It extends from the dorsal brain commissure and connects through two pygidial neurite bundles with the ventral nerve cord (Figs. 2a, c, 3f, 6a, b, 7i). In *Polygordius appendiculatus* there is likewise a connection of the main dorsal neurite bundle to the brain [46]. The number and arrangement of the main peripheral longitudinal neurite bundles varies considerably among annelid representatives [41, 46] and might reflect their mode of life rather than their phylogeny.

In the long biramous segments of *G. oculata* there are numerous circular neurite bundles serially arranged along the body wall of each segment, whereas the short biramous segments exhibit a smaller number of rather thick circular neurite bundles that are associated with the noto- and neuropodia (Figs. 2, 7a). A similar arrangement of circular neurite bundles is also found in vestimentiferan siboglinids. In the large segments (vestimentum and trunk) there are numerous circular neurite bundles, while only one pair of circular neurite bundles is present in each of the tiny opisthosomal segments [85]. Thus, in *G. oculata,* and likely all oweniids, the number and arrangement of circular neurite bundles is defined by the length of the segments.

In general, the number of circular serially arranged neurite bundles branching off from the ventral nerve cord is highly variable in Annelida [41]. The number of circular neurite bundles per segment is probably constrained by the overall morphology that is adapted to its particular ecology. We suppose that it is not beneficial to argue what is the ancestral condition, several pairs [43] or numerous circular neurite bundles per segment [34, 53].

### Pygidial innervation

For the first time three clusters of numerous somata were found in the pygidial lobes in oweniids (Figs. 6a, b). In this study, we could not confirm that these ICC-positive somata accumulations are organized as ganglia [49]. However, ultrastructural studies are needed to confirm if there is a neuropil surrounded by cell bodies (cortex) that commonly defines a ganglion.

In annelids the innervation of the pygidium has only been poorly studied. In species bearing pygidial appendages there have been serotonin-lir and FMRFamide-lir somata reported [86–88], while in the orbiniid *Nainereis,* that lacks pygidial appendages, mostly FMRFamide-lir somata are present (Victor Starunov, personal communication). In *G. oculata* the pattern of the pygidial neurites is congruent with annelids that lack pygidial appendages. The presence of the somata accumulations together with the ciliary epidermis of the pygidial lobes (Figs. 1g, 3f) suggests that they might form the sensory centers of the pygidium.

## Conclusions

This is the first study of the oweniid nervous system by means of immunocytochemistry and confocal laser scanning microscopy. The “medullary brain commissure” of *Galathowenia oculata* (and possibly all oweniids) is a neuroanatomical feature only known from one other annelid taxon, Echiura. Both taxa exhibit a single brain commissure, a burrowing mode of life and lack head appendages. Circumesophageal connectives and the ventral nerve cord of *G. oculata* are medullary organized and most likely a derived feature within the annelids. The only accumulations of ICC-positive somata are associated with eyes and pygidial lobes and do not form ganglia. The single ventral nerve cord is paired along its entire length. In *G. oculata* the neuronal elements are distributed differently in the segments of different length. Long segments exhibit numerous serially arranged circular neurite bundles and the medullary ventral cord. In short biramous segments ICC-positive somata and circular neurite bundles are metamerically arranged. Although Oweniidae (together with Magelonidae) appears as a sister clade to the rest of Annelida in contemporary phylogenomic analyses, many features of its nervous system listed above greatly deviate from that found in most annelids as well as from most putative annelid sister groups, suggesting that these are derived rather than plesiomorphic annelid conditions that evolved along the line leading to the Oweniidae.

## Methods

### Collection and fixation of *Galathowenia oculata*

Adult *Galathowenia oculata* typically inhabit muddy sediments. The specimens were collected by dredging from about 30–40 m depth in the Kandalaksha Bay near the Krestovy Islands (N 66°32.63, E 033°13.19) in the vicinity of the White Sea Biological Station, Lomonosov Moscow State University, Russia, in September 2012 and June-July 2013. Worms were extracted from their tubes, anesthetized in 5.1 % MgCl_2_ in seawater, and fixed in 4 % paraformaldehyde (PFA) in 0.1 M phosphate-buffered saline (PBS, pH 7.4) for 4 h at room temperature (RT) or overnight at 4 °C, rinsed thrice in 0.1 M PBS and stored in 0.1 M PBS with 0.1 % NaN_3_ at 4 °C for further scanning electron microscopy (SEM) and immunocytochemical studies. Specimens for transmission electron microscopy (TEM) were fixed in 3 % glutaraldehyde in 0,1 M PBS with 3 % sucrose and later postfixed in 1 % osmium tetroxide for one hour.

#### Immunolabelling

The following staining procedures and antibodies were used: DAPI (Invitrogen, Eugen, OR, USA), monoclonal mouse anti-acetylated α-tubulin (Sigma-Aldrich, St. Louis, MO, USA) with Alexa Fluor 488-labeled secondary antibody directed against mouse (Invitrogen); polyclonal rabbit anti-serotonin (Zymed, San Francisco, CA, USA) with either an Alexa Fluor 568-labeled secondary antibody directed against rabbit (Invitrogen) or with an Alexa Fluor 633-labeled secondary antibody directed against rabbit (Invitrogen); polyclonal rabbit anti-FMRFamide (Chemicon, Temecula, CA, USA) with TRITC-labeled secondary antibody against rabbit (Sigma-Aldrich). Various combinations of double and triple stainings were used for the species investigated.

Prior to whole-mount staining of specimens, unspecific binding sites were blocked in PBT (PBS containing 10 % Triton-X-100) and 4 % normal goat serum (Invitrogen) overnight at 4 °C. One or two primary antibodies were applied (mixed 1:1 with double concentrations; diluted 1:500 in PBT with 4 % NGS) and incubated for 48 h on a shaker at room temperature (RT). Afterwards, the samples were washed several times with PBT and one or two secondary antibodies were applied in a dilution of 1:300 in PBT with 1 % NGS for 12 h on a shaker at RT. Thereafter, animals were incubated for 1 h in DAPI in PBS in a 1:40 dilution and rinsed several times in PBS before specimens were mounted in Fluoromount G (Sigma-Aldrich) or glycerol between 2 coverslips to allow scanning of animals from both sides. Negative controls were performed by omitting the primary antibody and rendered no signal. Total number of used specimens in the study was 37 (including 12 specimens as controls).

#### Immunocytochemical staining of vibratome sections

Prior to sectioning, pieces of adults of *Galathowenia oculata* were rinsed in 0.1 M PBS over 20 min at 4 °C. Samples were embedded in a gelatine-ovalbumine solution and stored in 10 % formaldehyde in 0.1 M PBS overnight at 4 °C. Thereafter, samples were rinsed in 0.1 M PBS for 4–5 h, then sectioned in transverse, parasagittal and parafrontal 50–150 μm thick sections using a Leica VT1200S vibratome (Leica Microsystems, Wetzlar, Germany). Vibratome sections were stained in the same manner as whole-mounts, but for permeabilization the PBT contained 2 % Triton X-100. Incubation in primary antibodies was reduced to 24 h. Dilution of antibodies was increased to 1:600 for primary antibodies and 1:800 for secondary antibodies. Animals were mounted in Elvanol [89] on glass slides.

#### Confocal microscopy

All samples were investigated with a Leica TCS SP5 confocal microscope (CLSM) (Leica Microsystems, Wetzlar, Germany) or a Nikon A1 CLSM (Nikon Corporation, Tokyo, Japan). Leica or Nikon imaging software was used to generate optical sections with a Z-step size of 0.1–1.5 μm which were digitally merged to yield maximum and average projection images. Depth-coded Z-stack images, obtained with the Leica software provided with the confocal microscope, follows the area of the spectral light, with the uppermost structures appearing blue and the more distant ones red. All images were further processed with Adobe Photoshop CS6 (Adobe Systems, San Jose, CA, USA) to adjust contrast and brightness. Drawings were generated with Adobe Illustrator CC.

#### Electron microscopy

For scanning electron microscopy *Galathowenia oculata* specimens were postfixed in 1 % OsO_4_ and dehydrated in an ascending ethanol and acetone series, critical point dried and then sputter coated with platinum-palladium. Specimens were examined with a JEOL JSM scanning electron microscope (JEOL Ltd., Tokyo, Japan).

For transmission electron microscopy specimens were dehydrated in an alcohol series using standard protocols and thereafter polymerized for 20–24 h at 50 °C and embeded in Epon. The block was trimmed to the object and sectioned into ultrathin (50–70 nm) sections using a Leica EM UC7 ultramicrotome (Leica Microsystems, Wetzlar, Germany). Ultrathin sections were mounted on slot grids and mesh grids, contrasted with 2 % uranyl acetate- and 4 % lead citrate-solution and examined using a Zeiss Libra 120 electron microscope (Zeiss, Oberkochen, Germany).

#### Documentation of adult specimens

Images of adults were taken using a Leica DFC420С (5.0MP) camera mounted on a Leica DM2500 dissecting microscope. Images were processed with Leica Application Suite software.
